# Vaccine Therapy with Special Reference to So-Called Surgical Tuberculosis

**Published:** 1911-03

**Authors:** Lewis M. Gaines

**Affiliations:** Atlanta, Ga.


### VACCINE THERAPY WITH SPECIAL REFERENCE TO SO-CALLED SURGICAL TUBERCULOSIS.

By Lewis M. Gaines, A. B., M. D., Atlanta, Ga.

When Wright's work on opsonins, and his method of treating infections were first given publicity, there sprung forth as if by magic an enormous literature, devoted largely to optimistic therapeutics. Surgeons began to tremble for the future, and the latter days of the scalpel seemed to not a few dreamers close at hand. Laboratory workers began to see their workshops crowded with practitioners eager for the last opsonic index that the next dose of vaccine might be curative and not toxic. Internists beheld the approach of a new era in which the hypodermic needle replaced the knife and the horrors of operation were averted.

Experience, that bitter teacher, has dispelled many of these dreams. Few clinicians consider longer the opsonic index to be a necessary guide in the administration of vaccines. Many of the infections which vaccines held out such glorious hopes of overcoming, have proven refractory to this method. Our vaunted knowledge of the biologic intricacies of the human mechanism has received many a rude jolt. In spite, however, of these discouragements, progress has been made in the treating of certain forms of infection with proper vaccines, in proper doses at proper intervals.

It is of importance to carefully distinguish between a vaccine and a serum. A serum is always obtained from the blood of another animal than the one receiving it, and contains protective substances because the animal had been rendered immune to some particular infection. On the other hand, a vaccine is "any chemical substance, which, when introduced into the organism, causes there an elaboration of protective substances." These protective substances are elaborated in the infected organism in the case of a vaccine, introduced into it from without in the case of a serum. Hence the principle is fundamentally different, and the terms vaccine therapy, and serum therapy cannot be used interchangeably, as is often erroneously done.

Practically, vaccines consist of an emulsion of dead bacteria, carefully standardized, in order that the dose may be properly regulated. I shall not, in this brief paper detail the various infections wherein specific vaccines have proved of value, but proceed at once to deal with certain forms of tubercular infections, wherein vaccines derived from the tubercle bacilli have proved curative.

There are at the present time a very large number of different tuberculins, each one championed by a band of adherents. I have used in my work, for therapeutic purposes, Koch's Bacillen Emulsion. This form of tuberculin consists of an unheated fifty per cent. gycerin emulsion of living, virulent, pulverized tubercle bacilli containing 5 milligrams of solid substance in each cubic centimeter. The coarser particles are removed by centrifugalization. It is my belief that the undiluted emulsion should be obtained from a reputable house, and the dilutions constantly made up fresh. Many competent authorities are of the opinion that the high dilutions do not keep their strength longer than three or four weeks. Some manufacturers put out serial dilutions which have been made up for months. I do not consider this to be justifiable. I make up my low dilutions every few weeks, and my high dilutions at the time of administration.

Two forms of tuberculosis, which until recent years have been considered surgical affections have in many instances proved amenable to vaccine therapy. I desire to briefly call your attention to these forms of protean disease, and give two illustrative cases, as a type of the good results which may be obtained from this method of treatment.

The first of these forms is tubercular cervical adenitis, a condition which in former days was usually called scrofula. Until a very few years ago, a case of this kind usually underwent an experience somewhat as follows: Upon visiting the family physician, on account of gradually enlarging masses in the neck, the latter after a more or less vigorous massage, would conclude with a prescription of iodine painted over the glands externally, and perhaps some form of iodine internally, or if the patient were of a certain age, cod liver oil. Week by week, in spite of this treatment, the glands would increase in size, and at various localities about the face and neck, or perhaps in the axilla, new ones would appear. Finally, over a prominent mass the skin would become reddened, and the area become sore. This time, surgery was invoked, and the suppurating gland opened, and perhaps curetted. Then for weeks or months in one or more places there were foul running sores. Finally, if the patient recovered, it was to be disfigured for life. If the family physician was not too conservative, he may have advised a radical operation for removal of the glands early in the infection before suppuration took place. In this case, of course much time and discomfort would be saved, but still there would be disfigurement, and always the possibility, fairly strong, of a recurrence.

Upon the advent of vaccine therapy, it was found that tubercular adenitis frequently responded to this method of treatment. Anyone who has had the pleasure of seeing the enlargements gradually melt away week by week, and the patient recover without a scar of any description, and with a fair assurance that a recurrence is unlikely, will henceforth always give this method a thorough trial. I do not wish to be misunderstood. There are cases which do not improve under vaccine therapy. There are far advanced cases in which a certain amount of surgery must be done in connection with the tuberculin injections. But I wish to emphasize that a very great deal depends upon the way in which the vaccine is given.

I always start with a dose not larger than .0001 mg. This is preferably injected hypodermically between the shoulder blades. After four days if there has been no reaction, a second dose is given, usually .0002 mg. Then at intervals of four, five, six and, finally, seven days, a gradually increasing dose is given until a dose of .001 mg. is reached. Above this amount I rarely go, as it appears unnecessary, and may prove injurious. A reaction is an occurrence to be most scrupulously avoided. If it should take place, longer interval must be permitted, and the first subsequent dose, substantially diminished and an increase very gradually again achieved. Haste is impossible in this form of treatment, and patients should be clearly given to understand the duration and significance of the time element.

The following is a case I have selected because of its severity. M. S., age 14, a young girl reared in a comfortable and refined home, amid hygienic surroundings. Her cervical adenitis was over a year's duration, and she had had the usual experience. The condition had been temporized with, until she presented four or five foul running sores, a large amount of cicitricial red tissue, and numerous glandular enlargements on both sides of the neck, in both anterior and posterior triangles, under the jaw, and in front of the ear. In fact, wherever there was a gland in the neck or on the face, it appeared to be more or less enlarged, while in one axilla there was beginning enlargements which were quite tender. To briefly summarize, she was treated with vaccines as indicated, and after four months all suppuration had ceased, and was replaced by almost healed granulations, practically all of the enlargements had greatly diminished, or altogether disappeared. The axillary glands disappeared very early in the treatment. Her general health also greatly improved, a gain of fifteen pounds in weight being achieved. A slight but persistent afternoon temperature also early disappeared. She returned to her home in a distant city in this improved condition, which, I hear, has continued.

The second form of tuberculosis, usually considered surgical, is affections of the genito-urinary system. I desire to comment at this time exclusively upon renal tuberculosis. At the last meeting of the American Association of Genito-Urinary Surgeons at Washington, this matter was quite thoroughly discussed. Dr. F. E. Gardner of New York, in a paper upon this subject declares that there have been 83 cases of renal tuberculosis treated with tuberculin, of which 18 were called cured, 30 benefitted, and 25 unimproved. These statistics are, however, misleading, as the details of many of the cases were incomplete. He states that from the entire literature upon the subject we are only justified in drawing the conclusion that tuberculin raises the defensive power of the body. Whenever the defensive power is normally sufficient to cope with the morbid process, the reinforcement it receives from tuberculin is useful. The general consensus of opinion was favorable to the results of vaccine therapy, but not in favor of substituting it for operation, where operation held out hope of benefit.

From a limited number of cases, it is eminently foolish to attempt deductions. I make none, but simply present for consideration the following case:

J. B. R., farmer, aged 53, kindly referred to me by a physician in Anniston, Ala. Family history negative. Past History —Has never been very strong. One night about twenty years ago he passed two jagged stones from the urethra, accompanied by bleeding and some pain. Previous to this occurrence, he had had kidney colic on the left side, lasting 18 hours. There have been no more attacks of this character.

Present illness has been really of some years' duration. For some time has been suffering from pain and tenderness over the bladder, and extending up the left side and into the back, which is quite weak. Gets up two or three times at night to urinate. Urinates frequently through the day, often with considerable tenesmus. Has never passed blood. Is off six pounds from his normal weight, which is about 130 pounds. Has had no night sweats, fever or cough.

Physical examination revealed tenderness over the pubes, and over the left kidney, and along the left sacro-iliac articulation. The heart and lungs were normal, and nothing of interest to note further, except the examination of the urine. Before coming to me, two specimens of urine, one a catheterized specimen, had been examined by a pathologist in Birmingham, and on both occasions tubercle bacilli found. I examined a third specimen, and found a few tubercle bacilli, in addition to a small amount of albumin, and a moderate sediment, largely composed of pus.

This patient was immediately put upon tuberculin. The injections were continued 5 months. The improvement was steady from the outset. The pain and discomfort on urinating gradually disappeared entirely, and tubercle bacilli disappeared from the urine. He gained ten pounds in weight, and what is more important, gained remarkably in strength, which for some years had been greatly diminished. He was discharged in better health, according to his statement, than he had been for years. I saw this patient some months after his discharge, and he reported that he was still in excellent health. Whether or not this man will remain cured is another problem. He is apparently cured, and a treatment which for months even will relieve a patient of distressing symptoms is worth while.

I shall not dare to draw conclusions, but I do venture to assert that in vaccine therapy we have a weapon well worthy of trial in a large percentage, certainly, of these cases of so-called surgical tuberculosis. If the method proves a failure, it certainly has done no harm. I believe, however, that in experienced hands, administered with care, a very large percentage of cases will derive a maximum of benefit from its application.

823 Candler Bldg.

*Discussion of the paper of Dr. L. M. Gaines*:

Dr. H. P. Cole.—My experience with treatment in surgical tuberculosis is limited, but I have been very much interested in the writings of other men. Most of this work has been done in England, but the American doctors are also making great progress along that line. The doctor speaks of the different kinds of tuberculin, I think these could be summed up into two kinds, the old and the new. There are a great many exponents for tuberculin, while there are equally as many against it. Some physicians claim that for tuberculosis it is equal to digitalis in heart trouble. After a resume of eighty-four cases, one physician stated that in cases that are inoperatable, tuberculin can be used to advantage, and after operations where all the diseased tissue has not been removed, it is most effective. One physician now gives tuberculin by mouth with favorable results. After a resume of 61 cases, another physician advises the use of tuberculin. One physician reports 14 operations followed by tuberculin, where all recovered. I am glad the doctor called attention to the large number of new vaccines now on the market, and believe that we should be very cautious about taking up these things until we are sure about their merit. We should leave this vaccine treatment alone, unless, through men who are educated in the work, and are so situated that they can watch these patients for six or eight months. I think proper diagnosis is very necessary for the effective work of tuberculin.

Dr. Gaines, in closing.—I wish to emphasize further one point I mentioned, i. e., the large number of vaccines on the market. Some months ago I wrote one of the most prominent houses in this country, who put out a large number of vaccines, asking if they could justify their claim for their tuberculin. In reply to this, I received a two page letter from which I found that they could not justify themselves. I, therefore, wish to warn physicians against the indiscriminate use of these different tuberculins.

